# Morbidity and Mortality After Surgical Management of Tibial Plateau Fractures in Octogenarians

**DOI:** 10.5435/JAAOSGlobal-D-21-00109

**Published:** 2021-10-27

**Authors:** Tom G. Pollard, Puneet Gupta, Theodore Quan, Pradip Ramamurti, Safa C. Fassihi, Alex Gu, James DeBritz

**Affiliations:** From the Department of Orthopedic Surgery, George Washington Hospital, Washington, DC.

## Abstract

Because the geriatric population continues to increase in number, the incidence of geriatric tibial plateau fractures in octogenarians is projected to increase. The functional and physiological status varies within this population. The purpose of this study was to compare the complications after surgical management of tibial plateau fractures in patients aged 65 to 79 years with those in patients aged 80 to 89 years.

**Methods:**

In this retrospective cohort study, data were collected from the National Surgical Quality Improvement Program database for the years 2006 to 2018. The Current Procedural Terminology codes and International Classification of Diseases codes were used to identify all tibial plateau fractures that were treated with open reduction and internal fixation. Patients were divided into two groups: 65- to 79-year-old group and 80- to 89-year-old group. Primary and secondary outcomes were studied and included the 30-day mortality. Univariate and multivariate analyses were done with a statistical significance set at *P* < 0.05.

**Results:**

In total, 718 patients with tibial plateau fractures who underwent open reduction and internal fixation were included in this study. Of these, 612 were aged 65 to 79 years, and 106 were aged 80 to 89 years. On multivariate analysis, patients aged 80 to 89 years were at increased risk of postoperative anemia requiring transfusion (odds ratio 2.83; 95% confidence interval 1.37 to 5.84; *P* = 0.005) and extended length of hospital stay (odds ratio 2.72; 95% confidence interval 1.64 to 4.51; *P* < 0.001) in comparison with patients aged 65 to 79 years.

**Conclusion:**

In appropriately selected octogenarian patients, surgical management of tibial plateau fractures was associated with greater risks of transfusion and longer hospital stay. However, comparisons of the rates of late complications and reoperations remain unknown.

Tibial plateau fractures are most common in patients aged 30 to 60 years,^1^ often resulting from higher energy mechanisms of injury such as a pedestrian struck, fall from height, or motor vehicle collision.^1,2^ These fractures are often managed operatively except when minimally displaced.^3^ Nonsurgical management may also be indicated in the setting of multiple medical comorbidities making surgery too high risk, although this is rare in the younger population that typically sustains this injury.^4^ Recently, as the population ages, tibial plateau fractures are becoming more common in the elderly population and make up approximately 8% of all total fractures in those older than 65 years.^5^ When displaced, open reduction and internal fixation (ORIF) is the standard of care for these injuries, with the primary goal being restoration of the mechanical axis of the extremity and articular surface congruity.^3^ Geriatric tibial plateau fractures present unique challenges when managed operatively because they may exhibit a high degree of comminution, extensive surrounding soft tissue damage, and a bicondylar pattern despite lower energy mechanisms.^6,7^ These more complex injuries lend to longer surgical times, often requiring multiple surgical approaches, which may further increase risk for perioperative complications. Despite the added complexity, literature has shown clinical outcomes to be relatively good in this population. However, the functional and physiological status varies within the geriatric population.^5,7,8,9,10^ A better understanding of the potential complications as they relate to increasing age is critical because these fractures are becoming more common. The purpose of this study was to compare the complications after ORIF of tibial plateau fractures in patients aged 65 to 79 years with those in patients aged 80 to 89 years.

## Methods

This retrospective cohort study was conducted with data obtained from the American College of Surgeons National Surgical Quality Improvement Program (ACS-NSQIP) database for the years 2006 to 2018. The ACS-NSQIP is a national database that contains patient data from more than 600 participating institutions. These data are collected by trained clinical reviewers and include information such as preoperative demographics, risk factors, perioperative events, and postoperative complications for patients undergoing surgical procedures.^11^ Many studies have demonstrated high interrater reliability with data collection using this database.^12,13^ In orthopaedic surgery, the ACS-NSQIP database is widely used to track the clinical course of individual patients.^14-16^

### Patient Selection

Current Procedural Terminology codes 27535 and 27536 and various International Classification of Diseases, Ninth Revision and Tenth Revision, codes were used to identify all tibial plateau fractures that were treated with ORIF. Patients were excluded if they were younger than 65 years. Two patient cohorts were defined in this study: patients aged 65 to 79 years and patients aged 80 to 89 years.

### Baseline Patient Characteristics and Postoperative Outcomes

Patients' demographics and clinical characteristics data that were collected from the database included age, sex, race, body mass index (BMI), and American Society of Anesthesiologists (ASA) class. The primary outcome studied was the 30-day mortality. The secondary outcomes included superficial or deep surgical site infections, organ space infections, wound breakdown, pneumonia, unplanned intubation, pulmonary embolism, acute renal failure, urinary tract infection, bleeding requiring transfusion, myocardial infarction, deep vein thrombosis, sepsis, stroke, extended length of hospital stay, readmission, and revision surgery.^17^

### Statistical Analysis

Univariate and multivariate analyses were conducted using the Statistical Package for the Social Sciences (SPSS; Version 26) software. Using bivariate analysis, patient data on demographics, postoperative complications, and clinical characteristics were analyzed between the two study cohorts using Pearson chi-squared test and one-way analysis of variance where appropriate. A multivariate logistic regression analysis was done to adjust for sex, race, BMI, and ASA class to determine which postoperative complications differed between the two cohorts. Multivariate analysis results were reported as odds ratios (ORs) with 95% confidence intervals (CIs). A *P* value of <0.05 was the cutoff value for statistical significance in this study.

## Results

A total of 718 patients with tibial plateau fractures treated with ORIF were included in the final analysis after the exclusion criteria was applied, with 612 (85.2%) patients in the 65- to 79-year-old cohort and 106 (14.8%) patients in the 80- to 89-year-old cohort. Patients aged 80 to 89 years were more likely to experience an ASA class of III or IV fracture (*P* = 0.002) compared with those aged 65 to 79 years. The two cohorts did not differ significantly in sex, race, or BMI (Table 1).

**Table 1:** Demographic Features and Clinical Characteristics of 65- to 79-year-old and 80- to 89-year-Old Cohorts With Tibial Plateau Fracture Cohorts

	65- to 79-Year-Old Cohort (N = 612)	80- to 89-Year-Old Cohort (N = 106)	*P* Value
Age^a^ (yr)	70.29 ± 4.08 (65-79)	84.03 ± 2.96 (80-89)	<0.001^c^
Sex^b^			0.081^d^
Men	171 (27.9%)	21 (19.8%)	—
Women	441 (72.1%)	85 (80.2%)	
Race^b^			0.742^d^
White	437 (81.7%)	77 (83.7%)	—
Black	32 (6.0%)	7 (7.6%)	
Hispanic	52 (9.7%)	6 (6.5%)	
American Indian	3 (0.6%)	1 (1.1%)	
Asian	11 (2.1%)	1 (1.1%)	
BMI^a^ (kg/m^2^)	28.94 ± 6.45 (18.81-56.53)	28.13 ± 7.36 (19.24-68.59)	0.245^c^
ASA classification^b^			0.002^d^
ASA I (normal health)	27 (4.4%)	2 (1.9%)	—
ASA II (mild systemic disease)	289 (47.2%)	32 (30.2%)	
ASA III (severe systemic disease)	266 (43.5%)	61 (57.5%)	
ASA IV (severe systemic disease that is life-threatening)	29 (4.7%)	11 (10.4%)	
ASA V (moribund)	1 (0.2%)	0 (0.0%)	

ASA = American Society of Anesthesiologists, BMI = body mass index

a The values represent the mean and the SD, with the range indicated in parentheses.

b The values represent the number of patients, with the percentage indicated in parentheses.

c Analysis of variance.

d Pearson chi-squared test.

On univariate analysis, pneumonia (2.8% versus 0.7%; *P* = 0.035), requirement for blood transfusion (16.0% versus 5.7%; *P* < 0.001), and extended length of hospital stay (71.4% versus 44.8%; *P* < 0.001) were significantly increased in the 80- to 89-year-old cohort compared with those in the 65- to 79-year-old cohort. However, the 30-day mortality was not significantly different between the two cohorts (zero versus 0.3%; *P* = 0.556) (Table 2).

**Table 2:** Comparison of Mortality and Morbidity Between the 65 to 79 years Old and 80 to 89 years Old Tibial Plateau Fracture Cohorts, Univariate Analysis

30-Day Outcomes	65- to 79-Year-Old Cohort (N = 612)	80- to 89-Year-Old Cohort (N = 106)	*P* Value
Surgical complications (%)			
Superficial surgical site infection	0.7	0.9	0.740
Deep surgical site infection	0.7	0.0	0.404
Organ space infection	0.7	0.0	0.404
Wound disruption	0.2	0.0	0.677
Nonsurgical complications (%)			
Pneumonia	0.7	2.8	0.035
Unplanned intubation	0.7	0.0	0.404
Pulmonary embolism	0.5	0.0	0.470
Acute renal failure	0.5	0.0	0.470
Urinary tract infection	2.5	5.7	0.070
Requiring transfusion	5.7	16.0	<0.001
Myocardial infarction	0.2	0.0	0.677
Deep vein thrombosis	2.1	0.9	0.417
Sepsis	0.7	0.9	0.740
Stroke	0.3	0.0	0.556
Extended length of stay (>3 d)	44.8	71.4	<0.001
30-d readmission	4.9	8.6	0.202
Revision surgery	2.5	0.9	0.332
Death	0.3	0.0	0.556

On multivariate analysis, after controlling for sex, race, BMI, and ASA, the 80- to 89-year-old cohort no longer had significantly increased rates of pneumonia (OR 3.71; 95% CI 0.69 to 20.04; *P* = 0.127). However, being aged 80 to 89 years was independently associated with a significantly increased likelihood of requiring blood transfusion (OR 2.83; 95% CI 1.37 to 5.84; *P* = 0.005) and extended length of stay (OR 2.72; 95% CI, 1.64 to 4.51; *P* < 0.001) (Table 3).

**Table 3:** Multivariable Logistic Regression Analysis Adjusted for Sex, Race, BMI, and ASA Class

80-89-Year-Old Cohort (versus 65- to 79-Year-Old Cohort) 30-Day Outcomes	OR	95% CI	*P* Value
Pneumonia	3.71	0.69-20.04	0.127
Requiring transfusion	2.83	1.37-5.84	0.005
Extended length of stay (>3 d)	2.72	1.64-4.51	<0.001

ASA = American Society of Anesthesiologists, BMI = body mass index, CI = confidence interval, OR = odds ratio

## Conclusion

Geriatric tibial plateau fractures have become more common as the population ages, representing a substantial portion of fractures sustained in those older than 65 years.^5^ In the management of geriatric fractures, the treating orthopaedic surgeon must consider the variation in the functional and physiological status within this patient population. A better understanding of the potential complications of surgical management of these injuries because they relate to increasing age is vital when counseling patients regarding surgical versus nonsurgical management. The purpose of this study was to compare the complications after surgical treatment of tibial plateau fractures for patients aged 65 to 79 years with those aged 80 to 89 years.

Our study found that octogenarian patients were at a markedly increased risk for postoperative anemia requiring transfusion (OR 2.83; 95% CI, 1.37 to 5.84; *P* = 0.005) and extended length of hospital stay (OR 2.72; 95% CI, 1.64; *P* < 0.001) compared with patients aged 65 to 79 years after undergoing ORIF of a tibial plateau fracture. To our knowledge, no studies have considered octogenarians as a separate cohort when assessing rates of postoperative complications in tibial plateau fractures treated with ORIF. Despite this, multiple studies have shown reasonably good clinical outcomes when these injuries are managed operatively. For instance, a study by Su et al^9^ found that 87.2% of patients with an average age of 67 years had excellent or good clinical results after being managed surgically. Similarly, Hsu et al^8^ reviewed 22 tibial plateau fractures managed surgically in patients with a mean age of 66 years and found restoration of range of motion with flexion to 120° and no extension lag in all but one patient and no complications requiring further surgery. Biyani et al^18^ retrospectively reviewed 32 patients with a mean age of 71.7 years and found 23 good or excellent clinical and radiographic outcomes after tibial plateau ORIF. Similarly, Frattini et al^19^ retrospectively reviewed 49 patients with a mean age of 72 years and found clinical and radiographic outcomes to be satisfactory or better in 75.5% and 59.1% of patients, respectively. In addition, Horne et al^20^ found comparable outcomes and patient satisfaction between operatively and nonoperatively managed tibial plateau fractures in a population with a mean age of 73.6 years, potentially making a case for nonsurgical management in appropriately selected patients.

Because these injuries are often managed operatively and outcomes are generally satisfactory or better, surgeon knowledge of outcome data among this cohort is important to adequately counsel geriatric patients considering surgical management of a tibial plateau fracture. We believe our study is the first to specifically consider geriatric tibial plateau fractures with a focus on octogenarians. Conclusions regarding postoperative morbidity and mortality after surgical management of other octogenarian fractures were mixed. Noordin et al^21^ found that octogenarian patients who underwent dynamic hip screw fixation for intertrochanteric femur fractures had a higher rate of postoperative complications (25%) and 1-year mortality (22%) than those younger than 80 years (4% and 8%, respectively). Similarly, in a study by Zhao et al^22^ age greater than 82 years was found to be an independent risk factor of in-hospital postoperative pneumonia after surgical management of intertrochanteric femur fractures. In addition, Matityahu et al^23^ found that octogenarians who sustained operatively managed pelvic fractures had a higher risk of mortality and complications than the nonoctogenarian geriatric population. Moreover, a prospective observational study by Barbosa et al^24^ of 182 patients showed that an increase in age by 1 year within the geriatric population increased the OR for mortality by 4% in patients with a operatively managed proximal femur fracture. Goch et al^25^ compared the outcomes of those aged 55 to 69 years with those of septuagenarians and octogenarians after ORIF for proximal humerus fractures. The authors found that there was no significant difference between both groups regarding revision surgery rate, complications, and mean Disabilities of the Arm, Shoulder, and Hand score.^25^ Furthermore, Ma et al^26^ found surgical management of femoral neck and intertrochanteric femur fractures in octogenarians to have no increased risk of morbidity but did have a markedly increased risk of mortality compared with nonoctogenarians. In addition, Gil et al^27^ found that patients older than 80 years had neither an increased mortality rate nor a higher rate of complications compared with those aged between 65 and 79 after surgical management of an ankle fracture. Finally, an increase in perioperative complications in octogenarian patients was demonstrated across multiple surgical disciplines, including cardiac, colorectal, and head and neck surgery.^28,29,30,31^

This study has several limitations. Because this is a retrospective database study, information regarding surgical indications, baseline function, preexisting arthritis, medical risk factors, fracture pattern, and details of surgical treatment were not included. ASA classification is included, however, which serves as a surrogate for medical comorbidities and takes patient age into account. In particular, because of the inherent limitations of the NSQIP database, the complications were recorded only up to 30 days postoperatively. Therefore, this study was able to comment only on the risks of short-term complications. Because of this, late sequelae such as arthrosis and revision surgeries were not included in this analysis. Inherent to database studies is the potential for errors in data input and collection. However, the potential bias in data collection should affect each cohort equally and, thus, should not change our results markedly. Moreover, some hospitals do not participate in the NSQIP, and although it is a large database, our sample size is relatively small, with only 106 operatively managed tibial plateau fractures in the octogenarian cohort. This likely reflects the relatively low frequency of such injuries in this population. Furthermore, it is also likely that many patients with moderate or severe preexisting arthrosis or those with minimal fracture displacement were managed nonoperatively. In addition, patients with significant preexisting medical comorbidities or other risk factors might have been managed nonoperatively despite having a fracture pattern that is typically considered surgical. Despite these limitations, this is a reasonably well-powered study that is the first to specifically characterize morbidity and mortality after ORIF for geriatric tibial plateau fractures with a focus on octogenarians.

In conclusion, important physiological and functional variation exists within the geriatric population. This study provides treating surgeons with outcomes data that can be used when counseling patients on anticipated benefits and risks of surgical versus nonsurgical management of tibial plateau fractures. In appropriately selected octogenarian patients, surgical management of tibial plateau fractures is associated with greater risks of transfusion and longer hospital stay. However, comparisons of late complications and revision surgeries remain unknown.
